# A rigorous approach for selection of optimal variant sets for carrier screening with demonstration of clinical utility

**DOI:** 10.1002/mgg3.148

**Published:** 2015-04-23

**Authors:** Cynthia Perreault-Micale, Jocelyn Davie, Benjamin Breton, Stephanie Hallam, Valerie Greger

**Affiliations:** Good Start Genetics, Inc.237 Putnam Avenue, Cambridge, Massachusetts, 02139

**Keywords:** *BLM*, bloom syndrome, carrier screening, next-generation DNA sequencing, recessive disorders, variant classification

## Abstract

Carrier screening for certain diseases is recommended by major medical and Ashkenazi Jewish (AJ) societies. Most carrier screening panels test only for common, ethnic-specific variants. However, with formerly isolated ethnic groups becoming increasingly intermixed, this approach is becoming inadequate. Our objective was to develop a rigorous process to curate all variants, for relevant genes, into a database and then apply stringent clinical validity classification criteria to each in order to retain only those with clear evidence for pathogenicity. The resulting variant set, in conjunction with next-generation DNA sequencing (NGS), then affords the capability for an ethnically diverse, comprehensive, highly specific carrier-screening assay. The clinical utility of our approach was demonstrated by screening a pan-ethnic population of 22,864 individuals for Bloom syndrome carrier status using a *BLM* variant panel comprised of 50 pathogenic variants. In addition to carriers of the common AJ founder variant, we identified 57 carriers of other pathogenic *BLM* variants. All variants reported had previously been curated and their clinical validity documented, or were of a type that met our stringent, preassigned validity criteria. Thus, it was possible to confidently report an increased number of Bloom’s syndrome carriers compared to traditional, ethnicity-based screening, while not reducing the specificity of the screening due to reporting variants of unknown clinical significance.

## Introduction

Genetic disorders with severe phenotypes are generally rare, but may have a relatively high incidence and correspondingly high carrier frequency in certain populations. For example, the Ashkenazi Jewish (AJ) population has an elevated incidence for a number of disorders. Caucasians share a high incidence of cystic fibrosis and spinal muscular atrophy with the AJ population, while other populations have an increased incidence of other diseases. Carrier screening for up to 19 AJ disorders is recommended by the American Congress of Obstetricians and Gynecologists (ACOG), the American College of Medical Genetics and Genomics (ACMG), and various AJ societies (ACOG committee opinion 2004; Monaghan et al. 2008; http://www.victorcenters.org, http://www.jewishgeneticdiseases.org, last accessed 22 September 2014).

The first AJ population carrier screening program, instituted for Tay-Sachs disease in the early 1970s, reduced the incidence of the disease 10-fold (Kaback et al. 1993). The success of this program has led to the current recommendations for carrier screening programs for other disorders. It is estimated that about 1 in 3.3 AJs is a carrier for at least one of 16 so-called AJ disorders (Scott et al. 2010). In individuals of 100% AJ ancestry, testing for a relatively small number of founder variants have historically detected >95% of carriers (ACOG committee opinion 2004). Traditional carrier screening assays specifically target selected ethnic groups, such as AJs, and typically assess only common, founder variants within a gene. Cost considerations, restrictive technologies and lack of knowledge about variant distribution in nontarget populations limited this design. While screening for population-specific variants may yield high detection rates in respective target populations (e.g., the AJ), the detection rates outside of these ethnicities, or in patients of mixed ethnic background, are often suboptimal. Intermarriage with other ethnic groups is occurring more often in the AJ community (http://www.pewforum.org/2013/10/01/jewish-american-beliefs-attitudes-culture-survey/, last accessed 4 March 2015), and thus, this carrier screening approach has become increasingly unsatisfactory, both within the AJ community and outside.

To address this challenge, we have developed a NGS carrier screening assay that has been validated technically (Umbarger et al. 2014) and clinically (Hallam et al. 2014) and reliably detects a variety of sequence variants within specific genes. NGS represents a rapid and comprehensive method to screen entire genes, and provides the opportunity of finding a much larger set of sequence variants across many ethnic groups, including entirely novel variants. However, interpreting the large number of variants observed by this approach remains a significant challenge. Since no follow-up testing is available for genetic carrier screening, specificity is an important factor and variants of unknown significance (VUS) should not be reported. We therefore established a rigorous process that could be used to select an optimal variant set for population and sequencing-based carrier screening for a variety of recessive disorders that is both highly sensitive and highly specific for pathogenic variants. We describe stringent rules for variant classification using three categories of evidence, and, using Bloom syndrome as an example, outline the steps for creating a highly curated variant panel.

The clinical utility of our process is illustrated here through the creation of a highly curated variant panel for Bloom syndrome and applying it to a large pan-ethnic population. Bloom syndrome is phenotypically characterized by short stature, sun sensitivity, increased risk of cancer, distinctive facial features, and sometimes learning disabilities, an increased risk of diabetes, chronic obstructive pulmonary disease (COPD), and recurrent infections. Both men and women with Bloom syndrome can also experience reduced or absent fertility (http://ghr.nlm.nih.gov/condition/bloom-syndrome, last accessed 23 September 2014). Bloom syndrome is one of the disorders with increased incidence in AJs, but has also been observed in other ethnicities.

We provide data to show that our unique panel of 50 *BLM* sequence variants is effective for screening a pan-ethnic population and superior to the traditional carrier screening approaches currently available. Our methodology is broadly applicable to NGS-based screening for a variety of disorders, and can serve as a guideline for the development of NGS-based carrier screening panels for an unlimited number of additional disorders.

## Materials and Methods

### Collection of relevant literature

Literature searches were performed in PubMed (http://www.ncbi.nlm.nih.gov/pubmed, last accessed 12 September 2014). Several search strings were customized for each gene. Gene and/or disorder name and the words mutation or variant were used as keywords for all genes. We also searched for publications authored by leading researchers in the fields, and articles detailing carrier screening guidelines. Search results were inspected by curators, and full length articles were obtained for all relevant matches. In addition, we searched the internet and the resource provided by HGVS (http://www.hgvs.org/dblist/glsdb.html, last accessed 12 September 2014) for Locus-specific variant databases.

### Variant database construction

Curators carefully reviewed all articles and all potentially pathogenic variants (variants observed in a patient with the relevant phenotype) were entered into our database. All variants detectable by the standard NGS protocol, namely single-nucleotide substitutions or insertions/deletions not exceeding 10 bp that are located in exons or within the first 10 bp of an intron were collected. In addition, all variants with known or potential clinical relevance were included, even if they could not be detected by our standard NGS protocol. Excluded were known very rare variants not amenable to detection by NGS, gross chromosomal rearrangements, such as translocations and inversions, and variants with insufficient quality or literature to support their validity or genomic location. Known benign variants (such as high-frequency variants) were usually not recorded.

Variants were named according to HGVS-recommended nomenclature (http://www.hgvs.org/mutnomen/, last accessed 12 September 2014). All available data associated with each variant was collected. Entries included common aliases, the approximate number of alleles observed for the variant, information relevant for classification, such as summaries of experimental and genetic data, populations in which the variant was detected, nature of the sequence change (missense, nonsense, synonymous, in-frame indel, frameshift indel, etc.), and all publications referencing the variant.

An independent researcher verified the name, position, and nucleotide change for each variant considered for inclusion on the panel. The disorders, genes and respective reference sequences used are presented in Table1.

**Table 1:** The disorders, genes responsible, and corresponding OMIM, CCDS, and NM numbers are presented for all the variants that are included in our carrier screening panel

Disorder	OMIM entry	Gene	CCDS#	RefSeq (NM number)
Cystic fibrosis	#219700	*CFTR*	CCDS5773.1	NM_000492.3
Usher syndrome, type 1F	#602083	*PCDH15*	CCDS7248.1	NM_033056.3
Familial hyperinsulinism	#256450	*ABCC8*	CCDS31437.1	NM_000352.3
Canavan disease	#271900	*ASPA*	CCDS11028.1	NM_000049.2
Maple syrup urine disease, type A	#248600	*BCKDHA*	CCDS12581.1	NM_000709.3
Maple syrup urine disease, type B	#248600	*BCKDHB*	CCDS4994.1	NM_000056.3
Bloom syndrome	#210900	*BLM*	CCDS10363.1	NM_000057.2
Usher syndrome, type III	#276902	*CLRN1*	CCDS3153.1	NM_174878.2
Dihydrolipoamide dehydrogenase deficiency	#246900	*DLD*	CCDS5749.1	NM_000108.3
Fanconi anemia group C	#227645	*FANCC*	CCDS35071	NM_000136.2
Glycogen storage disease, type 1A	#232200	*G6PC*	CCDS11446.1	NM_000151.3
Tay-Sachs disease	#272800	*HEXA*	CCDS10243.1	NM_000520.4
Familial dysautonomia	#223900	*IKBKAP*	CCDS6773.1	NM_003640.3
Mucolipidosis, type IV	#252650	*MCOLN1*	CCDS12180.1	NM_020533.2
Niemann-Pick disease, type AB	#257200, #607616	*SMPD1*	CCDS44531.1	NM_000543.4

### Classification of variants

Classification was limited to identifying variants that are considered pathogenic with high confidence, in other words, those variants with compelling evidence that the variant does cause the phenotype in question. Classification criteria described here apply to recessive Mendelian disorders and highly penetrant variants with relatively large effects. Classification criteria were established following recommendations in the literature (Richards et al. 2008; Maddalena et al. 2005; Strom 2005).

A variant was classified as pathogenic if it was (a) observed in a patient with the appropriate phenotypes AND (b) met at least one of the following criteria in the following three evidence-based categories:

1. *Sequence-based evidence*. The variant in question must be expected to be truncating. This includes frame shift indels (deletions or insertions where the number of base pairs is not a multiple of three), nonsense variants, variants at invariant splice site positions (+1G; +2T; −2A; −1G), read-through variants (variation in the normal stop codon), and variations of the initiation codon (ATG).
2. *Experimental evidence*. A variant is considered to be pathogenic if there is unambiguous experimental evidence that it has a significant impact on gene or protein function. Assays must be comparable across laboratories and give clear and easy to interpret results. Variants suspected to affect splicing are relatively straightforward to validate experimentally by demonstrating the presence of an aberrant splice product in high abundance, for instance by transfecting minigenes into cell lines not expressing the normal transcript (Sosnay et al. 2013; Scott et al. 2012; Sharma et al. 2014; Fernandez-Guerra et al. 2010). Loss of protein function is often more difficult to demonstrate, and robust and meaningful assays are not always available. Typically, wild-type and mutant alleles are expressed *in* *vitro* in cells that lack the activity of the protein to be tested. The activity of the mutant is then compared to the WT. Pathogenic variants are expected to show no, or very low, residual activity.
3. *Genetic evidence*. A variant was classified as pathogenic based on genetic evidence if it was a founder variant, or if there was statistical evidence showing the variant was significantly more frequent in affected individuals than in controls (MacArthur et al. 2014). We used Fisher’s exact test with log-transformed *P*-values. A log score of ≥1.3, implicating a significance at *α* = 0.05, was used as cut-off value. The allele frequencies in patients are derived from studies performing variant screening in sizeable (ideally >50) patient populations. Those studies frequently provide inadequate or no controls. We therefore used the data collected by the 1000 genomes project to establish an upper limit of variant frequency in the general population.

**Figure 1:**
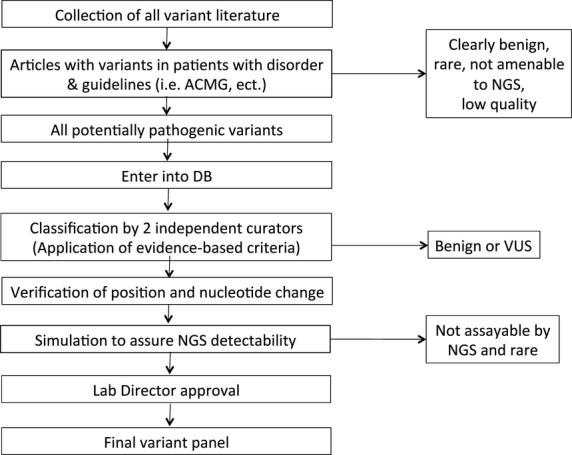
An overview of the process used to construct our final variant panels for carrier detection.

All classifications were performed by two independent curators. Figure1 provides an overview over the workflow.

### Simulations

Variants were simulated as described by Umbarger et al. (2014) to determine the rate of detectability in a set of at least 100 samples per variant. In order to be considered detectable by NGS, we required that a variant be detected in all samples in which it was simulated. Clinically relevant variants that were not detectable by NGS were assayed by an alternate technology.

### Study group

Study participants were 22,864 ethnically diverse individuals referred by fertility clinics from across the United States for routine carrier screening between April 2012 and February 2014. Informed consent processes were followed for each patient.

### Variant detection

Variants were detected by sequencing of the genes included in the panel. The NGS platform and NGS-based carrier screening protocol used has been previously described (Umbarger et al. 2014; Hallam et al. 2014). Briefly, this procedure involves multiplex target capture using tiled, molecular inversion probes followed by PCR-mediated incorporation of patient-specific molecular barcodes and Illumina (San Diego, CA) sequencing adapters. These products (representing the coding region and intron-exon borders of the genes investigated) were then sequenced using Illumina Hiseq2000 and 2500 instruments. Data were processed as described using a combination of open-source and internally developed tools for sample demultiplexing, short-read assembly and alignment, genotype calling, and functional annotation. Low quality calls and homozygous reference positions were filtered. Previously described alleles were identified using the in-house developed database of *BLM* gene variants. The initial phase of our study used standard Allele-Specific Primer Extension (ASPE) genotyping methods to detect *BLM* c.2207_2212delinsTAGATTC. This technique was subsequently replaced by the NGS detection method described above and all of the *BLM* variants were assayed by NGS technology only.

## Results

### Panel selection

Comprehensive variant panels for NGS-based carrier sequencing were selected for the following diseases (gene symbols are shown in parentheses, and in Table1): Canavan disease (*ASPA*), cystic fibrosis (*CFTR*), glycogen storage disorder, type 1a (*G6PC*), Niemann-Pick disease (*SMPD1*), Tay-Sachs disease (*HEXA*), Bloom syndrome (*BLM*), Fanconi anemia C (*FANCC*), familial hyperinsulinism (*ABCC8*), maple syrup urine disease, type 1A (*BCKDHA*) and type 1B (*BCKDHB*), Usher syndrome, type III (*CLRN1*), dihydrolipoamide dehydrogenase deficiency (*DLD*), familial dysautonomia (*IKBKAP*), mucolipidosis, type IV (*MCOLN1*), and Usher syndrome, type 1F (*PCDH15*). Variants in these genes are responsible for the first 15 diseases validated using the NGS platform.

An exhaustive compendium of known, potentially disease-causing variants in a gene is necessary to select a comprehensive carrier screening panel. For *CFTR*, such a collection exists in the Cystic Fibrosis Mutation Database (http://www.genet.sickkids.on.ca/Home.html, last accessed 23 September 2014). The CFTR database was used as a starting point for the variant collection for *CFTR* and supplemented with original data from the literature whenever available. For all other genes, we built our own collections through PubMed searches for published articles on variants implicated in each disorder. The first version of our panel described here includes articles indexed on Medline by March 2011. The only other locus-specific database (aside from *CFTR*) of sufficient quality for our purpose was the Fanconi anemia database (http://chromium.liacs.nl/LOVD2/FANC/home.php?select_db=FANCC, last accessed 23 September 2014), and it was mainly used for cross-checking purposes.

More than 1000 publications were reviewed for the first version of our panel. Over 2700 variants across all genes were entered in our variant database (VDB), annotated and evaluated, following the process outlined in Figure1. Using our stringent evaluation criteria, two-thirds of those variants did not have ample evidence to be considered pathogenic and were, thus, regarded as VUS, or benign. The remainder were classified as pathogenic with high confidence by two independent reviewers. All pathogenic variants then underwent quality control to verify that they had been recorded correctly. Computer simulations were performed to assess NGS detectability of variants as previously described (Umbarger et al. 2014). Rare variants not readily evaluated by NGS were excluded at this point. However, all clinically important variants were assayed by alternate methodologies if necessary for detection (Hallam et al. 2014).

Following this process, 976 variants were selected across 15 genes for the final Good Start Genetics (GSG) variant panel (first version, March 2011, see Table2). After a final vetting, variants were approved by the laboratory director and then included into the production database, or final panel. Table2 shows the number of variants selected for each disorder. To illustrate the steps taken, we describe below the variant selection for the Bloom syndrome gene (*BLM*) in detail. An equally robust process was followed up for each variant accepted into our final screening panel, that is, for all variants and all diseases for which we screen.

**Table 2:** The number of variants selected for the first version of our final carrier screening panel for each disorder

Disease	Gene	No. of variants
Bloom syndrome	*BLM*	50
Canavan disease	*ASPA*	41
Cystic fibrosis	*CFTR*	550
Dihydrolipoamide dehydrogenase deficiency	*DLD*	3
Familial dysautonomia	*IKBKAP*	2
Familial hyperinsulinism	*ABCC8*	64
Fanconi anemia group C	*FANCC*	24
Glycogen storage disease, type 1A	*G6PC*	66
Maple syrup urine disease, type 1A	*BCKDHA*	18
Maple syrup urine disease, type 1B	*BCKDHB*	20
Mucolipidosis type IV	*MCOLN1*	9
Niemann-Pick disease, type A/B	*SMPD1*	42
Tay-Sachs disease^1^	*HEXA*	67
Usher syndrome, type IF	*PCDH15*	15
Usher syndrome, type III	*CLRN1*	5

Includes two benign pseudodeficiency alleles.

### Specific example: bloom syndrome

A search using the string (BLM[Title/Abstract] OR Bloom Syndrome[Title/Abstract]) AND (mutation*[Title/Abstract] OR variant*[Title/Abstract]) yielded over 300 database matches in PubMed. Only nine articles described variants in human Bloom Syndrome patients and were utilized for variant collection, although others were useful for other purposes, such as experimental evidence classification. The fact that nearly all variants known at that time were found in patients enrolled in the Bloom Syndrome Registry and listed in the excellent review by German et al. (2007) greatly facilitated the process.

Articles were evaluated and variants entered into the database as per the protocol outlined. In total, our search yielded 77 *BLM* variants, of which 50 were eventually classified as pathogenic as described in more detail below and presented in Table3.

**Table 3:** The panel used for *BLM* carrier screening and the type of evidence used to classify the variant as pathogenic

cDNA Name	Protein name	Reason for panel inclusion	Effect of mutation	Reference(s)
c.1088-2A>G		Sequence-derived evidence	Splice site	German et al. (2007)
c.1090A>T	p.Arg364X	Sequence-derived evidence	Nonsense	German et al. (2007)
c.1284G>A	p.Trp428X	Sequence-derived evidence	Nonsense	German et al. (2007)
c.1346delC		Sequence-derived evidence	Frameshift	German et al. (2007)
c.1544dupA		Sequence-derived evidence	Frameshift	German et al. (2007), Kaneko et al. (2004)
c.1628T>A	p.Leu543X	Sequence-derived evidence	Nonsense	German et al. (2007)
c.1642C>T	p.Gln548X	Sequence-derived evidence	Nonsense	German et al. (2007), Antczak et al. (2013), Sokolenko et al. (2012)
c.1701G>A	p.Trp567X	Sequence-derived evidence	Nonsense	German et al. (2007)
c.1784C>A	p.Ser595X	Sequence-derived evidence	Nonsense	German et al. (2007)
c.1933C>T	p.Gln645X	Sequence-derived evidence	Nonsense	German et al. (2007)
c.1968dupG		Sequence-derived evidence	Frameshift	German et al. (2007)
c.2015A>G	p.Gln672Arg	Experimental evidence	Missense	Neff et al. (1999), German et al. (2007)
c.2074+1G>T		Sequence-derived evidence	Splice site	German et al. (2007)
c.2098C>T	p.Gln700X	Sequence-derived evidence	Nonsense	German et al. (2007)
c.2193+2T>G		Sequence-derived evidence	Splice Site	German et al. (2007)
c.2250_2251insAAAT		Sequence-derived evidence	Frameshift	German et al. (2007)
c.2254C>T	p.Gln752X	Sequence-derived evidence	Nonsense	German et al. (2007)
c.2406+2T>G		Sequence-derived evidence	Splice Site	German et al. (2007)
c.2407dupT		Sequence-derived evidence	Frameshift	German et al. (2007)
c.2488dupA		Sequence-derived evidence	Frameshift	German et al. (2007)
c.2506_2507delAG		Sequence-derived evidence	Frameshift	German et al. (2007)
c.2643G>A	p.Trp881X	Sequence-derived evidence	Nonsense	German et al. (2007)
c.2695C>T	p.Arg899X	Sequence-derived evidence	Nonsense	German et al. (2007)
c.2725C>T	p.Gln909X	Sequence-derived evidence	Nonsense	German et al. (2007)
c.275delA		Sequence-derived evidence	Frameshift	German et al. (2007)
c.2821C>T	p.Gln941X	Sequence-derived evidence	Nonsense	Amor-Gueret et al. (2008)
c.2923delC		Sequence-derived evidence	Frameshift	German et al. (2007)
c.3028delG		Sequence-derived evidence	Frameshift	German et al. (2007)
c.3118C>T	p.Gln1040X	Sequence-derived evidence	Nonsense	German et al. (2007)
c.311C>A	p.Ser104X	Sequence-derived evidence	Nonsense	German et al. (2007)
c.3164G>C	p.Cys1055Ser	Experimental evidence	Missense	Neff et al. (2011), German et al. (2007)
c.3223dupA		Sequence-derived evidence	Frameshift	German et al. (2007)
c.3255_3256insT		Sequence-derived evidence	Frameshift	German et al. (2007)
c.3261delT		Sequence-derived evidence	Frameshift	German et al. (2007)
c.3278C>G	p.Ser1093X	Sequence-derived evidence	Nonsense	German et al. (2007)
c.3415C>T	p.Arg1139X	Sequence-derived evidence	Nonsense	German et al. (2007), Adams et al. (2013)
c.3475_3476delTT		Sequence-derived evidence	Frameshift	German et al. (2007)
c.3510T>A	p.Tyr1170X	Sequence-derived evidence	Nonsense	German et al. (2007)
c.3558+1G>A		Sequence-derived evidence	Splice site	German et al. (2007)
c.3587delG		Sequence-derived evidence	Frameshift	German et al. (2007), Amor-Gueret. et al (2008)
c.3681delA		Sequence-derived evidence	Frameshift	German et al. (2007)
c.3727dupA		Sequence-derived evidence	Frameshift	German et al. (2007)
c.3847C>T	p.Gln1283X	Sequence-derived evidence	Nonsense	German et al. (2007)
c.557_559delCAA	p.Ser186X	Sequence-derived evidence	Nonsense	Kaneko. et al (2004), German et al. (2007)
c.581_582delTT		Sequence-derived evidence	Frameshift	Amor-Gueret et al. (2008)
c.582delT		Sequence-derived evidence	Frameshift	German et al. (2007)
c.772_773delCT		Sequence-derived evidence	Frameshift	German et al. (2007)
c.814A>T	p.Lys272X	Sequence-derived evidence	Nonsense	German et al. (2007)
c.98+1G>T		Sequence-derived evidence	Splice site	German et al. (2007)
c.2207_2212delinsTAGATTC		Genetic evidence	Frameshift	German et al. (2007), Scott et al. (2010)

c.2207_2212delinsTAGATTC was initially detected by genotyping methods.

*BLM* is a somewhat unusual gene because the vast majority of suspected disease-causing variants discovered to date are truncating (German et al. 2007) and thus could be classified with relative ease using sequence-based evidence. c.2207_2212delinsTAGATTC, which constitutes about 99% of *BLM* alleles in the AJ population, also has, as a founder variant, genetic evidence for classification. Only a few missense variants have been described, and all of them have been observed only a few times. To classify them, experimental evidence was required. Cells from patients with Bloom syndrome show an elevated rate of sister-chromatid exchange (SCE), thus variants were evaluated based on their ability to restore SCE to normal levels in *in* *vitro* assays with appropriate controls and performed with the variant in question in isolation (typically in transfected cell lysates). The assay is robust and well-established. In fact this methodology was used to initially map the *BLM* gene (Ellis et al. 1995). We classified two variants, c.2015A>G, p.Gln672Arg, and c.3164G>C, p.Cys1055Ser as pathogenic, because plasmid constructs containing those variants in transformed cell lines from Bloom patients failed to alter the frequency of SCEs (Neff et al. 1999).

26 variants did not meet our strict criteria for pathogenicity (data not shown). Some variants were clearly benign, even though reported in a Bloom syndrome patient. One example is c.3945C>T (p.Leu1315Leu). Its allele frequency of up to 20% (German et al. 2007) in the general population clearly speaks against pathogenicity. The missense variant c.3970C>T, p.His1324Tyr (German et al. 2007) did correct the high levels of SCE in a cell line and thus was considered not disease-causing. Other variants, such as c.1882+5G>A, seen once in a Bloom syndrome patient, did not have sufficient evidence of any kind to make a decision about pathogenicity. As more evidence becomes available, these VUS may eventually be classified.

The next step was to test whether pathogenic variants could be reliably detected by NGS. Simulations showed that one variant, c.2207_2212delinsTAGATTC was not be detected by NGS at the level of confidence required. Because of its clinical importance c.2207_2212delinsTAGATTC was assayed by standard ASPE genotyping initially. The final panel of the first version of our test for *BLM*, as shown in table3, consisted of 50 variants, 49 detectable by NGS, and one assayed byASPE. Subsequent improvements of algorithms allowed reliable detection of this variant by NGS, and c.2207_2212delinsTAGATTC is now part of our current panel of 50 *BLM* variants detectable by NGS.

### Testing of the variant selection process with *BLM* variants

We then applied our *BLM* variant panel to NGS-derived sequencing data using a cohort of 22,864 individuals. Not all of the research study participants were tested for c.2207_2212delinsTAGATTC, and so some of our clinical experience is based only on a subset of 10,701 patients who were also assessed for c.2207_2212delinsTAGATTC.

**Figure 2:**
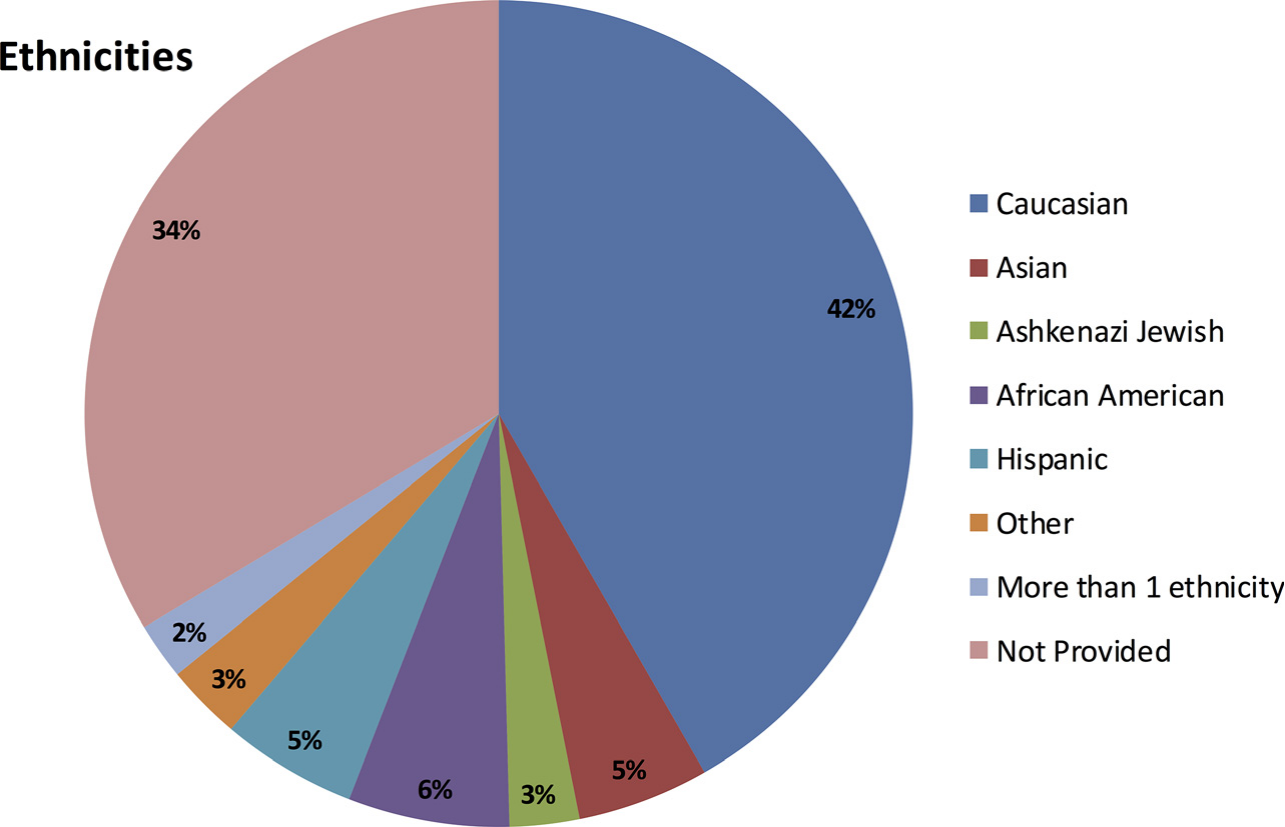
Ethnicity of the study population that included 22,864 individuals from fertility clinics within the United States.

66% of patients reported ethnicity, country of origin, or a combination of both. We used the following ethnic categories: Asian, African-American, AJ, Caucasian (including French Canadian), Hispanic, other (all that did not fall into one of the above categories), and more than one ethnicity. 439 individuals reported AJ as their only ancestry. Since Bloom syndrome is considered to be an AJ disorder, 183 additional participants that reported AJ plus one or more other ethnicity were reassigned to the AJ group. The majority of them (147 individuals) identified themselves as AJ plus Caucasian or specified a country of origin indicative of Caucasian ancestry, such as Germany or Russia. The other 36 reported one or more other additional ethnicities. Adding those 183 individuals to the AJ group raised the percentage of AJs in the overall population from 1.9% to 2.9%. Figure2 presents the ethnic distribution of our study group.

We found 39 carriers of 11 unique NGS-detectable, known pathogenic *BLM* variants among 22,864 patients. Table4 contains a list of those variants detected, the number of carriers, and their respective ethnicities in both the larger study population (22,864) and the smaller population in which the presence or absence of c.2207_2212delinsTAGATTC was determined. The most frequently seen variants are p.Gln548X (11 patients), p.Arg899X (seven patients), and p.Gln645X (five patients). Only two instances of p.Gln548X had been reported in the literature (German et al. 2007) at the time the variant panel was finalized. p.Gln548X was later found to be the most common pathogenic *BLM* variant in Slavic populations, with allele frequencies up to 0.6% (Antczak et al. 2013; Sokolenko et al. 2012). Eight variants were observed between one and three times. We did not observe c.2407dupT, the minor AJ variant.

**Table 4:** Carriers of known *BLM* variants from our panel and their respective ethnicities

cDNA Name	Protein name	No. of carriers (in 22,864)	No. of carriers (in 10,701)	Allele frequency (%)	Ethnicities of carriers	Allele frequency in ExAC (%)
c.2207_2212delinsTAGATTC		nd	6	0.0280	AJ3, NP3	0
c.1642C>T	p.Gln548X	11	5	0.0241	C8, NP3	0.0183
c.2695C>T	p.Arg899X	9	4	0.0197	C5, NP4	0.0066
c.1933C>T	p.Gln645X	5	1	0.0109	C3, A1, NP1	0.0041
c.2015A>G	p.Gln672Arg	3	1	0.0066	C2, NP1	0.0016
c.3847C>T	p.Gln1283X	3	2	0.0066	C2, NP1	0
c.2098C>T	p.Gln700X	2	0	0.0044	C2	0.0016
c.3415C>T	p.Arg1139X	2	1	0.0044	C1, NP1	0.0016
c.3164G>C	p.Cys1055Ser	1	1	0.0022	H+C1	0.0017
c.3261delT		1	0	0.0022	C1	0
c.3475_3476delTT		1	0	0.0022	C1	0
c.98+1G>T		1	0	0.0022	C1	0.0014

Two study populations are presented because not all individuals in the larger study population of 22,864 were assayed for the presence or absence of the c.2207_2212delinsTAGATTC variant. The allele frequency of variants present in the ExAC data set are shown in the last column. AA, African-American; C, Caucasian; NP, not provided; A, Asian; H, Hispanic; a + indicates 2 ethnicities reported.

Surveying entire genes using NGS not only allows detection of a much larger number of (predetermined) pathogenic variants, but also identifies novel, previously unreported pathogenic variants. Determining the clinical significance of the many novel, i.e., previously unreported, variants identified using NGS-based diagnostics tools remains one of the most challenging aspects of whole gene sequencing to date. However, truncating variants (as defined in the Materials and Methods section) are generally thought to disrupt gene function (Maddalena et al. 2005; Sosnay et al. 2013). Since in all the genes assessed here loss of function leads to disease, novel truncating variants observed in our study population are predicted to be pathogenic. We found 16 such truncating variants that have thus far not been described in the literature as observed in a Bloom syndrome patient. One of those variants was identified in three different individuals in our study population, the other 15 were found in one individual each.

Comparison to the Exome Aggregation Consortium (ExAC) data: We examined whether any known or novel variants we detected were also found in the ExAC data set (http://exac.broadinstitute.org, last accessed 5 March 2015) that includes 60,706 unrelated individuals without severe pediatric disease. The allele frequencies of the known pathogenic variants found in the ExAC data set are listed in Table4. Six of 16 of our “novel truncating” variants were also present in ExAC. c.3558+1G>T, detected three times in our study population (0.0066%) was seen three times in ExAC (0.0025%). Interestingly, there is a known pathogenic variant, c.3558+1G>A, at the same position, that was not observed in either study. The other five novel truncating variants we found that were also in the ExAC data set are c.320dupT and c.4076+1delG, both seen once in our study (0.0022%), and once in ExAC (0.0008%); c.357_358delAT, seen once in our study (0.0022%), and four times in ExAC (0.0033%); c.3875-2A>G, seen once in our dataset (0.0022%), and twice in ExAC (0.0016%) and; c.2923delC, seen once in our dataset (0.0022%), and five times in ExAC (0.0041%). Carrier rates: For calculating carrier rates, we used the subset of 10,701 patients from our clinical dataset, because these individuals were also assessed for the major AJ variant c.2207_2212delinsTAGATTC. We found a total of 28 Bloom syndrome carriers. Six (21%) carried c.2207_2212delinsTAGATTC, including three AJs, and three individuals that did not report ethnicity. Seven (25%) carried a novel variant. The overall carrier frequency was 1/382 for all types of variants, and 1/510, if novel variants are excluded.

## Discussion

Assessing the clinical significance of the many variants discoverable by sequencing-based tests is a rather daunting task, and must be done with rigor. Generally accepted and standardized approaches to variant analysis and interpretation are just beginning to emerge (Tavtigian et al. 2008; Maddalena et al. 2005; Duzkale et al. 2013; Brownstein et al. 2014; MacArthur et al. 2014). Many laboratories use five categories for variant classification: pathogenic, likely pathogenic, VUS, likely benign, and benign. Using five categories is necessary in a diagnostic setting; however, for carrier screening our focus was to identify variants that are considered pathogenic. Classification was also simplified by the fact that the only disorders considered here follow a recessive Mendelian mode of inheritance. In general, lack of data for determining pathogenicity for rare, nontruncating variants was the biggest problem. Literature reports, in particular older ones, are not reliable, and a significant portion of variants reported as disease-causing and contained in a variety of databases were later found to be benign (Bell et al. 2011). Computational methods predicting the impact of single base pair substitutions, while helpful in some settings, are not yet performing well-enough to be used on their own for clinical carrier screening (Hicks et al. 2011; Thusberg et al. 2011), and were not included in our evaluation at this point. Functional tests are often considered the optimal way to accurately predict the impact of those variants (Maddalena et al. 2005; Strom 2005), and were incorporated whenever robust assays were available. However, many rare variants remain unclassified and efforts like the CFTR2 project (Sosnay et al. 2013) that integrate clinical and molecular findings and systematically assay functional consequences will ultimately be necessary to solve this problem.

We have described a comprehensive procedure to select pathogenic variants for providing highly sensitive and specific clinical carrier screening. We use the same process to periodically update all of our variant panels and add newly published variants or to reclassify variants for which additional data has become available. The aim of our study was to considerably increase sensitivity of a carrier screening test as compared to traditional genotyping tests, yet to retain maximum specificity, as indicated for population carrier screening. We accomplished this by including as many variants across all ethnic groups as could be identified from the public domain, and then thoroughly vetting their pathogenicity and rigorously excluding all VUS. We also ensured that all clinically important variants for a gene were part of the screening panel, even if they were not detectable by NGS, our primary testing method. We then applied the resulting panel to a large, pan-ethnic group of individuals referred from fertility clinics across the U.S. and demonstrated, using *BLM* as an example, that we are indeed able to detect carriers with this panel at a rate that is within expectations. For *BLM*, in addition to the AJ variant c.2207_2212delinsTAGATTC, we found another three pathogenic variants that occurred multiple times and may represent the major *BLM* disease alleles in some populations. However, most variants were seen only once or twice. We also detected 16 truncating variants that had not previously been observed in Bloom syndrome patients, but are very likely to be pathogenic. Approximately a quarter of carriers in our data set had such novel truncating variants. Results for genes of the other AJ diseases were roughly comparable (data not shown), with one or a few “common” disease variants, and a long tail of variants observed only once, some of them novel. Many of the variants in our panel were private to a single individual. This supports previous population scale whole-exome sequencing studies which found that rare variants are enriched for damaging, pathogenic alleles (Marth et al. 2011; Li et al. 2010). It is not surprising that large scale population sequencing uncovers previously unknown disease-causing variants for rare recessive disorders. The majority of rare variants show little sharing between human populations (Gravel et al. 2011). This strongly suggests that comprehensive sequencing of known recessive disease genes and careful analysis of variants is the best approach to confidently discover carriers of genetic diseases. By contrast, if one were to only genotype previously characterized disease alleles, many carriers would go undetected. By definition, only a small number of patients are known for those disorders and a correspondingly small number of disease alleles have been studied. The few variants common in specific populations such as the AJ are known, but most of the rare pathogenic variants remain to be discovered. As more individuals are sequenced, the fraction of all novel variants (including pathogenic ones) decreases (Keinan and Clark 2012). Accordingly, most of the *CFTR* gene variants seen in our study, including very rare ones, had already been documented. However, novel pathogenic *CFTR* gene variants are still discovered sporadically.

This variant distribution also has an impact on selecting panels for population-based carrier screening. The fact that a sizable portion of disease alleles are very rare and many variants have not yet been described in the literature means that traditional genotyping approaches, which assay the most “common” disease alleles, will frequently miss carriers. In the example of *BLM*, 25 study participants (40% of all carriers) had a known pathogenic variant that, to our knowledge, was not included in any other commercial carrier screening test. An additional 18 individuals (28% of all carriers) had one of 16 different novel truncating variants. We conclude that in order to efficiently capture carriers, variant panels for pan-ethnic screening need to test for as many variants as possible.

The overall prevalence of Bloom syndrome is unknown, but for the AJ population it is estimated to be about 1 in 50,000 (http://ghr.nlm.nih.gov/condition/bloom-syndrome, last accessed 11 September 2014). From the carrier rates for pathogenic *BLM* variants observed in our clinical population, we would expect a range of 0.96 (without novels) to 1.7 (novels included) individuals per million to carry two disease alleles. Our carrier rates are in agreement with expectations for a very rare disorder, particularly if the following factors are taken into account: the tested set is enriched for individuals of AJ descent (as noted above, this population has a high carrier frequency); and one of the clinical features of Bloom syndrome is infertility in men and reduced fertility in women (http://ghr.nlm.nih.gov/condition/bloom-syndrome, last accessed 22 September 2014), therefore it is conceivable that carriers of *BLM* variants may experience suboptimal fertility, and *BLM* carriers may be overrepresented in patients referred from fertility clinics. Increased carrier frequency in patients needing assisted reproductive technologies has already been reported for cystic fibrosis (Tomaiuolo et al. 2011), a disorder with male infertility as a clinical component.

It is also possible that some variants have incomplete penetrance. It should be noted, however, that for *BLM* variants classified as pathogenic no homozygotes were observed in the ExAC data set and no homozygotes or compound heterozygotes were detected in our cohort. On the other hand, the presence of two pathogenic *BLM* mutations might be embryonic lethal in some cases.

Most importantly, our data indicate that using a comprehensive panel of rigorously evaluated variants for carrier screening a pan-ethnic population provides major advantages in terms of increased rates of carrier detection. Limited genotyping panels would have missed the carrier status of many of the individuals we identified because they were not carriers of the major ethnically specific common variants. In addition, the procedure we have described to select and stringently classify variants is broadly applicable. It can be used to select pathogenic variants for clinical carrier screening panels for a variety of recessive disorders, it provides assurance of clinical validity for the mutations in the panel, and it will result in more comprehensive variant detection compared to ethnic-based, nonsequencing methodologies.

## Conflicts of Interest

All authors are employees of Good Start Genetics, Inc., in Cambridge, MA 02139. In addition, all full-time employees have stock options in the company and hence are potential future shareholders in Good Start Genetics, Inc.
